# Influence of Solid Solutions on the Al2024 High-Temperature Deformation Behavior

**DOI:** 10.3390/ma16186251

**Published:** 2023-09-17

**Authors:** Oscar A. Ruano, Alberto Orozco-Caballero, Marta Álvarez-Leal, Fernando Carreño

**Affiliations:** Physical Metallurgy Department, CENIM-CSIC, Av. Gregorio del Amo 8, 28040 Madrid, Spain; ruano@cenim.csic.es (O.A.R.); alberto.orozco.caballero@upm.es (A.O.-C.); m.alvarez@cetemet.es (M.Á.-L.)

**Keywords:** mechanical properties, hot deformation behavior, stacking fault energy, solutes, solid solution, 2024 aluminum alloy

## Abstract

The mechanical properties of 2024 aluminum alloy were studied after two different tempers. The T351 temper (solution heat treatment, stress relief, and natural aging) leads to high hardness and toughness. A thermal treatment consisting of heat-treating at 280 °C for 48 h and slow cooling in a furnace, named TT temper, was performed to increase the precipitate size and their separation while minimizing the amount of solutes in solid solution, which produced the minimum hardness for an overaged Al2024 alloy and a lower tensile flow stress than for the T351 temper. The flow stress strongly decreases and the elongation to failure strongly increases for both materials above 300 °C. Differences in strain rate at a given stress in the power law regime at all temperatures for both tempers and compared with pure aluminum are attributed to the influence of solutes in solid solutions, affecting both the glide and climb of dislocations. However, the stacking fault energy, SFE, alone does not account for the hot deformation behavior. Thus, it is the synergistic effect of various solutes that affects the entire deformation process, causing a decrease of three or four orders of magnitude in strain rate for a given stress with respect to the pure aluminum matrix values.

## 1. Introduction

Aluminum alloys are important in many industries due to their lightweight, high corrosion resistance, and high strength-to-weight ratio. Despite the introduction of composite materials in the aeronautic industry, high-resistance aluminum alloys are still very important for structurally reliable components and fracture-resistant parts for airframes, fuselages, engines, and a vast number of other components [1,2].

The 2xxx series of aluminum wrought alloys and related alloys, having copper as the principal alloying element followed by magnesium, are especially important in the aeronautic industry and are nowadays subjected to extensive research [3,4,5,6,7,8,9,10,11,12,13]. These are heat-treatable alloys that can be hardened under certain conditions of heating and cooling and are primarily used for aircraft structures, fuselages, and the lower part of wings under tension [14,15]. In addition, they can be spot-welded. The effect of heat treatment in these alloys may be pronounced, increasing the strength of the fully annealed, named O temper, almost three times in the T3 temper. As usual, ductility diminishes as strength increases.

In general, the strengthening effect increases when the atomic radius of the solute is much larger than that of Al. However, the effect of adding multiple solutes is not necessarily additive. An important factor is the precipitation hardening effect, especially due to the extensive precipitation of second phases containing manganese. In the same line, Cu and Mg are appreciated because they also contribute, in solid solution, to the strengthening effect. The usual heat treatment involves solubilization at high temperatures followed by rapid cooling or quenching, provoking solute supersaturation. Then, aging treatments are carried out to produce fine precipitates. Such heat treatment leads to high strength at low temperatures by precipitation strengthening, while at high temperatures it provides a beneficial high strength by the combination of the remaining precipitates and solutes in solid solution. Additionally, depending on the characteristics of each solute, especially its size, two distinct solutes can attract or repel each other and can form different “Cottrell atmospheres,” so that dislocations can move unexpectedly slower because their combined effect can be non-linear or multiplicative, not precisely additive.

At high temperatures, T > 0.6 Tm (where Tm is the absolute melting temperature), the strain rate, $\overset{\cdot }{\varepsilon }$, has been observed to be related to the absolute temperature, T, and stress, σ [16]:

(1) $$\overset{\cdot }{\varepsilon }\;=\;f(s)\exp\;(-Q/\mathrm{RT})\;(\sigma /E){}^{n}$$

where f(s) = function of microstructure, E = average unrelaxed polycrystalline Young’s modulus, n = stress exponent, Q = activation energy for plastic flow, and R = universal gas constant.

The function f(s) represents mainly the influence of grain size, d, subgrain size, λ, and dislocation density, ρ. Over a certain temperature range, Q is constant and is related to a particular deformation mechanism.

Although time-dependent deformation of metals at high temperatures, above 0.6 Tm, is usually correctly described by a diffusion-controlled slip deformation mechanism (Equation (1)), other mechanisms may become important. Grain boundary sliding (GBS) and directional diffusional flow may, in fact, dominate deformation, especially in fine-grained materials. These three mechanisms are considered to operate independently, are thermally activated, are controlled by atom diffusion, and can be described by Equation (1).

In the case of the Al2024 alloys, the grain size is usually much larger than 20 μm, which is about the upper limit to activate GBS and diffusional flow mechanisms. Therefore, slip creep should be the controlling mechanism. This mechanism is usually described by the following equation [17]:

(2) $$\overset{\cdot }{\varepsilon }\;=\;A\;D_{L}/b^{2}\;(\sigma /E){}^{5}$$

where A is the constant for a given material, b is the Burgers vector, and D_L_ is the lattice self-diffusion coefficient.

This mechanism assumes that a moving dislocation, which is the origin of deformation, is held by an obstacle on its slip plane. However, the dislocation can climb by diffusional processes to a parallel slip plane. The climbing process permits the dislocation to glide on a new plane until it encounters another obstacle. This process is constantly repeated. This is a typical case of sequential processes, glide and climb, where the slowest of the processes controls the strain rate. Therefore, for Equation (2), the climb process is the one that determines the stress exponent, n = 5.

This equation is strongly influenced by the stacking fault energy (SFE, γ) of the materials. A stacking fault is an irregularity in the normal stacking sequence of the atomic planes. These irregularities have associated a certain energy, which is called stacking-fault energy. The width of these planes is determined by this energy, and therefore a low SFE corresponds to a wide stacking fault, causing difficulties in the mobility of dislocations in the material. Equation (2), therefore, has been changed to take account of the SFE in the following form [17,18,19,20,21,22]:

(3) $$\overset{\cdot }{\varepsilon }\;=\;A^{\prime }\;\gamma^{q}\;D_{L}/b^{2}\;(\sigma /E){}^{5}$$

where q is the stacking fault energy exponent that is usually equal to about 3–3.5 for a number of materials [17,18,19,20,21,22]. Therefore, Equation (3) assumes that the SFE has a strong influence on the flow resistance of the material (the A’ constant) and not on the stress exponent value. In other words, the SFE affects, at least, the glide part of the sequential process (glide + climb).

SFE is heavily influenced by many factors, especially the amount of alloying elements and solutes [23] and the valence-electron to atom ratio [24], and has been proven to have a profound influence on the deformation behavior of materials [19,25,26,27,28,29,30,31,32,33,34]. As a result, a decrease of various orders of magnitude in the strain rate with decreasing SFE has been observed. It has also been proposed that climb is strongly affected [35,36,37] so that as γ decreases, the rate of climb and cross-slip decrease correspondingly [25,33,36,37,38].

However, research conducted on Al2024 alloy prior to severe plastic deformation [39] shows no correlation with SFE during high-temperature deformation, as will be shown in this paper. This finding implies that the high-temperature creep behavior of this alloy, and to some extent, that of the Al2XXX family, cannot be accurately predicted on the basis of its composition. The aim of this study is to understand the high-temperature deformation behavior of the Al2024 alloy by comparing two extreme alloy tempers with pure aluminum and focusing on the influence of their solute atoms at high temperatures. As it will be shown, further analyses regarding the influence and interaction of the diverse solutes on the stacking fault energy of the alloy should be carried out to fully understand the hot deformation behavior of this and other alloys comprising several different solutes.

## 2. Materials and Methods

The 2024 aluminum alloy was received from Alu-Stock in the form of rolled plates, 3 mm in thickness, in the T351 temper [40,41]. The nominal chemical composition is provided in Table 1.

**Figure 1:**
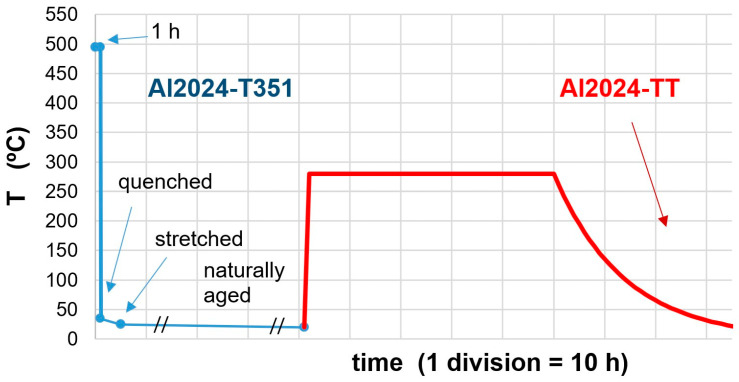
Thermal treatment history of Al2024-T351 (in blue, left) and TT (in red, right) alloys.

In the as-received state, the alloy exhibits high hardness values for this alloy system. The T351 temper is obtained by solution heat-treatment at 495 °C for 1 h, water quenching at a maximum of 40 °C, stress relief by controlled stretching, and then naturally aging. On the other hand, the minimum hardness state, named 2024-TT, was reached starting with the T351 material, heating at 280 °C for 48 h, and slow cooling in the furnace [42]. Figure 1 shows the thermal treatment history of both alloys (Al2024-T351 in blue, left, and Al2024-TT in red, right). The very slow cooling rate shown in the figure resulted in minimum hardness. This is the result of extensive overaging of precipitates and a strong reduction of solid solution atoms.

The microstructural characterization was carried out by means of optical microscopy (OM) and transmission electron microscopy (TEM). OM samples were prepared by conventional mechanical polishing and etched at room temperature with the Keller reactive, which consisted of a solution of 1.5% HCl, 1% HF, 2.5% of HNO_3,_ and 95% distilled water. TEM samples were electropolished until light detection at 15 V and 25 °C using a solution of 30% HNO_3_ and 70% CH_3_OH.

Instrumented ultramicroindentation was used to characterize the mechanical homogeneity of the Al 2024 alloy at room temperature using a diamond Berkovich-type indenter. The tests were carried out on Micromaterials Nanotest 600 equipment. The indents were obtained by applying a maximum load of 2 N. The detailed procedure can be found elsewhere [43].

Constant crosshead speed tensile tests (CCST) at an initial 10^−2^ s^−1^ were used to characterize the mechanical behavior of the alloy. In addition, tensile strain rate change tests (SRCT) ranging in strain rate from 10^−1^ to 10^−5^ s^−1^ were performed to characterize room temperature to high temperature (25–450 °C) behavior. The tests were performed using a universal Instron 1362 testing machine equipped with a four-lamp ellipsoidal furnace. Planar dog-bone tensile samples with 6.5 × 2 × 3 mm^3^ gage area dimensions were electro-discharge machined so that their longitudinal axis was parallel to the extrusion direction.

## 3. Results and Discussion

### 3.1. Microstructures

**Figure 2:**
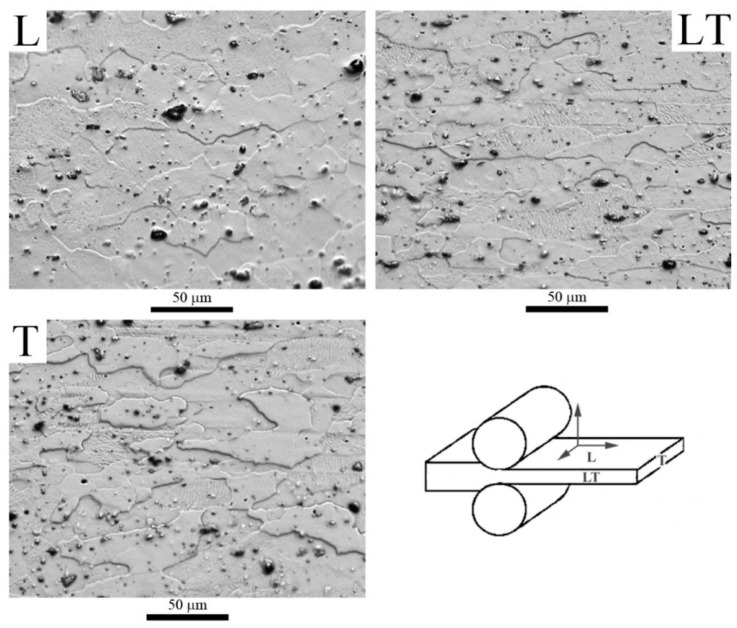
Optical micrographs of the as-received Al2024 alloy, T351 temper.

The microstructure of the as-received alloy in the T351 state was evaluated in the L, LT, and T (longitudinal, longitudinal-traverse, traverse) planes by means of optical microscopy (OM), as shown in Figure 2. The micrographs show elongated grains, especially in the LT plane, with a size between 50 and 150 µm on its major axis and between 20 and 40 µm on its minor axis. The precipitates, of different sizes and types, are distributed homogeneously throughout the sample. Additionally, we observed black particles of irregular morphology, ranging from 5–15 µm in size, which are called constituent particles. These particles consist of a complex composition rich in Fe, Mn, Si, and Al [44,45].

**Figure 3:**
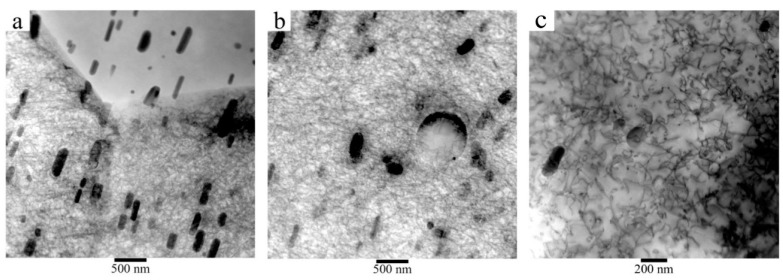
TEM micrographs of Al2024-T351. (**a**) Detail at a grain boundary, (**b**) precipitates inside a grain, and (**c**) showing tangled dislocations.

Figure 3 shows transmission electron microscopy (TEM) micrographs of the Al2024-T351 alloy with a second type of smaller precipitate. These precipitates are called hardeners and can have rod-like morphologies with a size between 50–500 nm on the major axis and 10–50 nm on the minor axis. The main composition of this type of compound is CuAl_2_ or CuMgAl_2_ [46,47]. Rod-morphology precipitates are oriented in the same direction and can cross grain boundaries, as shown in Figure 3a. Figure 3b shows a round-shaped precipitate about 600 nm in diameter. Figure 3c shows tangles of dislocations within a grain of the as-received alloy. This microstructure is totally different from that obtained after increasing the temperature up to 450 °C, where the hardening precipitates are dissolved in a coarse-grained matrix containing most elements in a solid solution [48].

**Figure 4:**
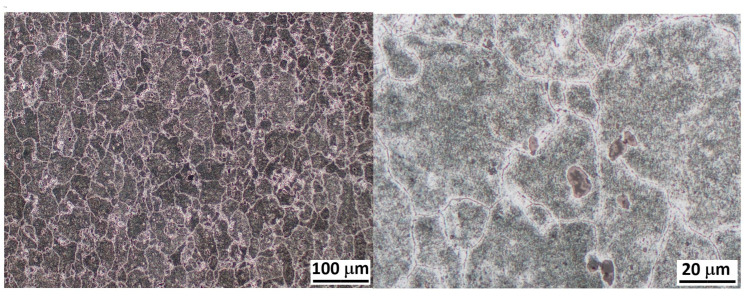
Optical micrographs of the Al2024 alloy under TT temper at two different magnifications.

Finally, Figure 4 shows optical micrographs of the Al2024 alloy under TT temper at two different magnifications with larger precipitates than in T351 (note the scale). This makes the separation between precipitates in the TT temper higher than in the T351 temper. The grains are less elongated compared to those of the T351 temper, with the grain size slightly larger. As observed in Figure 4, the precipitates of the TT temper are much larger than those of the T351 temper.

### 3.2. Ultramicrohardness

The microhardness of the Al 2024 alloy in two heat treatment tempers is given in Table 2. The as-received T351 temper presented the maximum hardness value, which drastically diminished after the heat treatment.

The high hardness value measured in the T351 temper is attributed to the presence of solid solution and, especially, the short distance between precipitates around 100 nm. After the TT temper, the solid solution content decreases, and the corresponding precipitates’ coarsening leads to a longer separation distance between them, as shown in Figure 4. Consequently, the possible dynamic processes such as nucleation and growth of new precipitates are minimized.

### 3.3. Tensile Tests at Intermediate and High Temperatures

**Figure 5:**
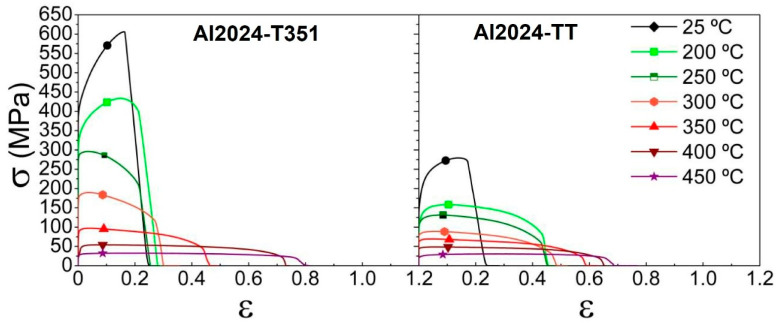
Stress-strain curves of Al2024 processed in two tempers and tested at an initial strain rate of 10^−2^ s^−1^ at temperatures from 25 to 450 °C.

Figure 5 shows stress-strain curves for the Al 2024 alloy for two different tempers at an initial strain rate of 10^−2^ s^−1^ at temperatures ranging from 25 to 450 °C. In general terms, flow stress values decrease with increasing testing temperatures for both tempers. Two different behaviors can be distinguished, one at low (high stresses and low ductility) and another at high (low stresses and high ductility) temperatures, with a transition temperature around 250–300 °C. The low-intermediate temperature regime (25–200 °C) shows very similar low tensile ductility values <25% for all T351 samples. In this regime, the stresses for the T351 material are considerably higher than for the TT material. At the highest temperatures, the stress is low and the ductility is high, with similar values for both tempers. The largest tensile ductility value and the lowest stress value are observed for both materials at 450 °C.

The mechanical parameters extracted from the curves in Figure 5 are given in Table 3. These parameters are yield stress (σ_0.2_), flow stress (σ), uniform elongation (e_u_), and elongation to failure (e_F_). A maximum elongation to failure of 122% and a maximum stress of 606 MPa are observed for the T351 material.

The evolution of mechanical properties for both tempers lies mainly in the evolution of the precipitates with the testing temperature, considering that both tempers present different precipitate natures and concentrations. It is worth mentioning that both tempers present an abrupt change in ductility and stress. At 300 °C, the Al2024-T351 still presents high stress values (190 MPa) and ductility (34%), while in the Al2024-TT state, the stress is lower (89 MPa) and the ductility is almost double (62%). This is attributed to the effect of the solutes and the precipitates in the T351 state, while in the TT temper, this effect is less important, with coarser precipitates. However, it is evident that there is a large drop in stress for both tempers due to the dissolution of the precipitates at high temperatures.

**Figure 6:**
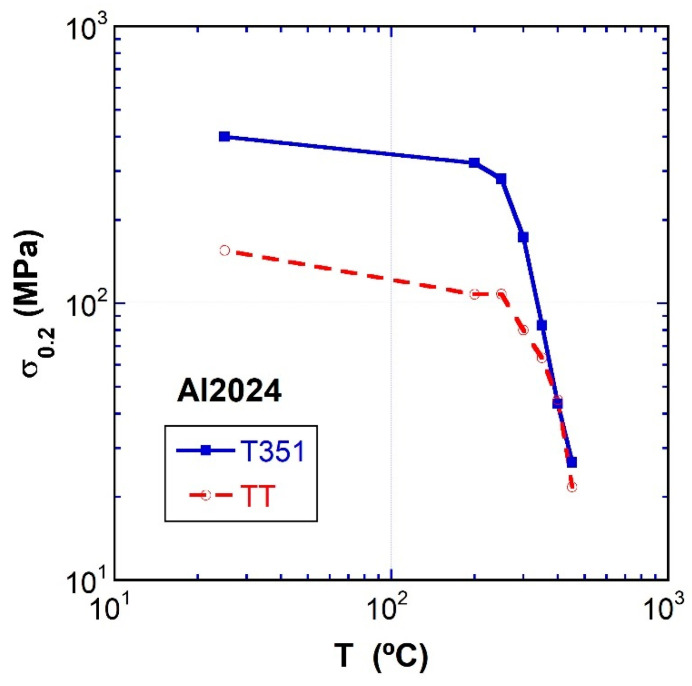
The flow stress, σ_0.2_, as a function of temperature at 10^−2^ s^−1^ for both tempers.

The flow stress, σ, as a function of temperature at 10^−2^ s^−1^ is represented in Figure 6 for both materials. The figure shows a moderate parallel decrease in stress with temperature from 25 to 250 °C. Above this temperature, the stress strongly drops for both materials towards similar values. It should be mentioned that at high temperatures, both time and temperature unavoidably dissolve the precipitates, which, at 450 °C, are residual and, therefore, do not influence the deformation behavior.

**Figure 7:**
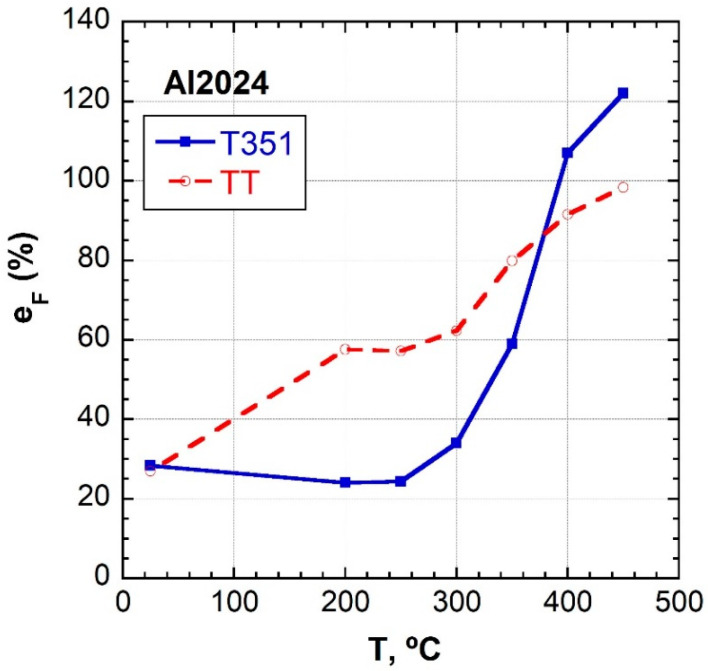
The elongation to failure as a function of temperature at 10^−2^ s^−1^ for both tempers.

Figure 7 shows curves of the elongation to failure at 10^−2^ s^−1^ as a function of temperature for the Al 2024 alloy for both temper conditions. In general terms, the ductility continuously increases with temperature up to a maximum temperature of 450 °C, except for the regime between 25–250 °C in the T351 temper, which could be attributed to the fine precipitation of the solid solution. At this temperature, the ductility values for the T351 temper are higher than for the TT temper. It can also be observed that in the range of 25–350 °C, the tensile ductility values of the T351 material are lower than those of the TT material.

### 3.4. Strain Rate Change Tensile Tests

**Figure 8:**
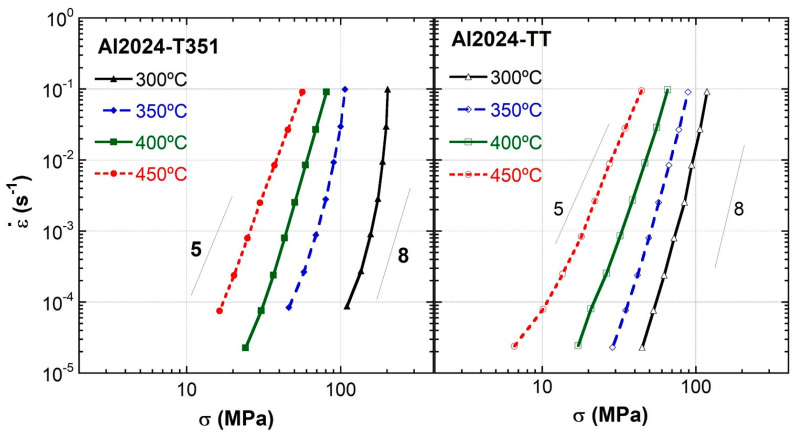
Double logarithmic scale representation of the $\overset{\cdot }{\varepsilon }$-σ pairs at different test temperatures of the Al 2024 alloy for both tempers.

Strain rate change tests at various temperatures were conducted to determine the stress exponents and the activation energies. This would allow us to gain information on the deformation mechanism at various temperatures for the Al 2024 aluminum alloy in the T351 and TT tempers. Figure 8 shows these tests at 300, 350, 400, and 450 °C.

Figure 8 shows a double logarithmic scale representation of the $\overset{\cdot }{\varepsilon }$-σ pairs at four different test temperatures of the Al 2024 alloy for both tempers. This figure shows that, for a given value of the strain rate, the stress decreases with temperature for both tempers. The material in the T351 temper is more resistant than in the TT temper at all temperatures, although this difference decreases as the temperature increases. It should be noted that the curve at 300 °C in condition T351 shows outstanding behavior because the fine precipitates still have a predominant effect, as mentioned previously. For this reason, the analysis of the high-temperature behavior was carried out without considering this temperature. At higher temperatures, the precipitates are dissolved, losing their importance and their effect and becoming the solid solution the relevant feature.

The value of the stress exponent is approximately five at the two highest temperatures and increases to about eight at the two lowest temperatures and high strain rates. The value of five corresponds to a mechanism of climb-controlled slip creep, which is common in pure materials and alloys with coarse grain sizes.

In relation to the activation energy for deformation, both temper materials show high values of about 200 kJ/mol. This value is higher than that corresponding to the self-diffusivity of aluminum. These high values are typical of reinforced aluminum alloys [49,50] and are indicative, on the one hand, that, with increasing time and temperature, the reinforcing precipitates present at the lowest temperatures dissolve and coarsen continuously, accelerating the decrease of flow stress and, thus, increasing the experimental or apparent activation energy value. On the other hand, a slight increase in the activation energy could be indicative of greater difficulty in the diffusion of the various interacting solutes in the aluminum matrix, which could lead to a lower effective diffusion rate compared to that of pure aluminum.

### 3.5. Deformation Mechanisms

A slow decrease of the UTS from room temperature up to about 250 °C and, from there, an abrupt decay at higher temperatures was shown in Figure 6. This contrasts with the behavior observed in other aluminum alloys, where a smooth decrease of the UTS is experienced up to high temperatures [17,51,52].

**Figure 9:**
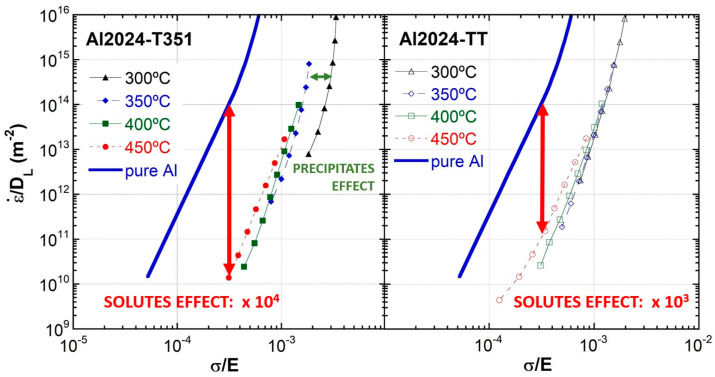
The strain rate normalized by lattice diffusivity versus stress normalized by Young’s modulus.

Figure 9 shows the stress data compensated by Young’s modulus at each temperature (σ/E) versus the strain rate compensated by the lattice self-diffusion coefficient ($\overset{\cdot }{\varepsilon }$/D_L_) obtained from strain rate change tensile tests for pure aluminum and for the as-received alloys in the precipitation states T351 and TT [17,35,51].

For pure aluminum, two zones are distinguished in the $\overset{\cdot }{\varepsilon }$/D_L_-σ/E curves. A region situated at $\overset{\cdot }{\varepsilon }$/D_L_ < 10^13^ m^−2^ values, known as the power law region, where data fit in a straight line with slope n = 5, and another region at $\overset{\cdot }{\varepsilon }$/D_L_ > 10^13^ m^−2^ values in which the slope increases progressively with increasing stress, named power law breakdown (PLB). Figure 9 shows that the stress values are higher for both Al 2024 materials compared to pure Al. The figure clearly shows that at 300 °C, the T351 material is more resistant. This was attributed to the fine precipitates in the T351 that were still present at this temperature. At 450 °C, both tempers show lower resistance compared to data at 400 and 350 °C, which is attributed to the fact that at 450 °C, most precipitates have already been dissolved and most elements are now in solid solution.

The pre-exponential constant for aluminum, A, that is included in the general equation for slip creep [17], Equation (2), is 2.5 × 10^12^. The values of the preexponential constants, considering an equation similar to Equation (2) that fits the data, for the Al2024 are 3.3 × 10^8^ and 2.5 × 10^9^ for the T351 and the TT materials, respectively. In other words, for a given stress, pure aluminum is 4 orders of magnitude less resistant in terms of strain rate than the T351 temper material and 3 orders of magnitude less resistant than the TT material.

Regarding the A constant, it is strongly influenced by the stacking fault energy, SFE. As mentioned before, the SFE influences the glide part of the sequential deformation mechanism (glide + climb) and the resistance of the material and is considered in Equation (3). This has been proven in Ni-Cu [17], where an increase in SFE from 20 to 200 erg/cm^2^ leads to an increase of $\overset{\cdot }{\varepsilon }$/D_L_ of almost 4 orders of magnitude. For this family of alloys, the SFE decreases with increasing amounts of solutes. In the case of pure aluminum, values of SFE varied from 200 to 350 erg/cm^2^ (350 mJ/m^2^) [53]. It should be noted that Al is a high stacking fault material compared to 80 mJ/m^2^ for Cu or 22 mJ/m^2^ for Ag [53].

Specific values of stacking fault energies for the Al2024 alloy have not been measured, but data on the influence of Cu and Mg on aluminum can be found in the literature [54,55]. The addition of 2% Cu in Al drops the SFE from 221 to 162 mJ/m^2^, and the addition of 2% Mg drops it to 144 mJ/m^2^. Therefore, the presence of elements in solid solution in Al could be relevant because increasing amounts of solutes decrease the SFE and increase the flow stress values [54]. Additionally, a synergic effect of several solute elements could be expected as the initial SFE value for Al is much higher than for other pure metals [53,56,57].

The mechanism explaining how the SFE affects deformation behavior is poorly understood at the moment, but a first approach points to the variation of the dislocation climb velocity. Other explanations involve the effect of dislocation constriction prior to climbing [37,58], vacancy emission from extended jogs [36], and the influence of SFE on the evolution of dislocation structure during deformation [35,58]. In addition, in the case of low SFE materials, cross-slip of dislocations should be difficult because the partials must first combine before the change of slip plane takes place.

The influence of SFE may partially explain the behavior of the three materials in Figure 9. In fact, the T351 material is likely to have a higher solute concentration than the TT material at all temperatures because it is easier to dissolve the fine precipitates before tensile testing. Thus, a lower SFE is also expected for the T351 material, the most resistant one.

Taking Equation (3) into account and assuming γ^3.5^, a drop in SFE of a factor of 1.58 (from 221 to 140 mJ/m^2^) causes a drop in the strain rate of 1.58^3.5^ = 4.96 times. This drop is too small to explain the 3 or 4 orders of magnitude decrease in strain rate. If the change in strain rate is only due to SFE, a decrease in SFE for aluminum of about ten times should be necessary, towards 20 mJ/m^2^, which has never been observed and is highly unlikely. Another approach to still considering the SFE important would be through its exponent, q, which could be larger than 3.5. However, even considering an exponent as high as 5, four times lower SFE would be necessary to lower by three orders of magnitude the strain rate at a given stress. Considering that the highest value of SFE in Al, reported by Muzyk [54], is 221 mJ/m^2^ and the lowest SFE values for some Al alloys are 90 mJ/m^2^ [54,59], the ratio is 2.46, which raised to the fifth power corresponds to an 89 times lower strain rate, far from the experimental values for the Al2024 alloys.

Therefore, we cannot explain the 1000–10,000 times lower strain rates of the Al2024 alloy than those of pure Al for the same given stress based only on stacking fault energy values. This is a clear indication that the solutes are influencing flow rates in additional ways.

It should be noted that the presence of solid solutions in the materials, even if they do not change the stacking fault energy, has an influence on the climb, which is the rate-controlling mechanism in slip creep. In this case, the diffusivities of the distinct solutes should be considered, especially if they influence each other mutually or synergistically, as could be expected by the coupling of solutes with dissimilar sizes, larger and smaller than the matrix atoms. This could be a similar case to that of magnesium WE54 alloy having large and small solutes [60,61] and showing extra resistance for the movement of dislocations and increased flow resistance.

Additionally, the presence of solid solutions in the materials, even if they do not change the stacking fault energy, has an influence on glide, controlling the mechanism of solute drag creep [47,60,62]. Therefore, the controlling deformation mechanism for the Al2024 alloy is climb-controlled slip creep at high temperatures, but the influence of solutes cannot be disregarded, taking into account that the higher the temperature, the higher the solute concentration. This is confirmed by the fact that the most resistant alloy, Al2024 T351, is the one with the largest amount of solid solution at any temperature, as its small precipitates could dissolve faster than the very gross and far apart precipitates obtained after the intensive TT thermal treatment. This treatment was meant to reduce its room-temperature hardness by overaging precipitates and diminishing the amount of solid solution.

In summary, the 2024 alloy high-temperature deformation behavior, although climb-controlled slip creep, is also affected by the amount of solutes in solid solution, lowering the effective diffusion rates for creep and influencing SFE. However, SFE is not solely responsible for the four orders of magnitude lower flow rates than for pure aluminum at 450 °C. The synergic effect of their solutes affects both glide and climb sequential processes for slip creep and lowers the effective diffusion rates for creep in this alloy. Further experimental and modeling research should be carried out to separate the different contributions, allowing for the prediction of the preexponential constants for Equations (2) and (3).

## 4. Conclusions

(1) Minimum hardness is reached for the Al2024 TT temper after a very slow cooling rate in the furnace, which results in coarse precipitates and reduction of solid solution atoms.
(2) The flow stress is higher for the T351 temper at all temperatures. A strong decrease occurs at temperatures higher than 300 °C for both materials. In contrast, the elongation to failure for the TT temper is higher than for the T351 temper, and a strong increase occurs for both materials above 300 °C.
(3) Differences in flow rate at a given stress are observed at all temperatures for the T351 and TT tempers and are especially high compared with pure aluminum, being about 3 and 4 orders of magnitude at the highest temperatures for which slip creep is the rate-controlling mechanism. At these high temperatures, most precipitates have dissolved, and the main reinforcing mechanism is attributed to solutes in solid solutions.
(4) The differences in high-temperature flow rate between Al2024 T351, Al2024 TT tempers, and pure aluminum are attributed to the influence of solutes in solid solutions on both glide and climb processes. Solutes in solid solutions affect stacking fault energies (SFE) and decrease both glide and climb rates for a given stress. However, the SFE alone does not explain the differences among the three materials.
(5) The synergic effect of various solutes and their influence on both glide and climb processes is important in this alloy and should be further investigated in other materials.

## Figures and Tables

**Table 1:** Nominal chemical composition of the Al 2024 alloy.

Element	Cu	Mg	Mn	Si	Fe	Zn	Ti	Cr	Al
wt. %	4.3	1.5	0.6	<0.5	<0.5	0.15	0.03	0.007	balance

**Table 2:** Average hardness values, H (GPa), of the Al 2024 in two temper conditions.

	Al2024-T351	Al2024-TT
H (GPa):	1.34 ± 0.02	0.65 ± 0.02

**Table 3:** Yield stress (σ_0.2_), UTS (σ_max_), uniform elongation (e_u_), and elongation to failure (e_F_) at different test temperatures of the alloy Al 2024 in two tempers and two precipitation states.

	T (°C)	σ_0.2_ (MPa)	σ_max_ (MPa)	e_u_ (%)	e_F_ (%)
Al2024-T351	25	398.8	606.2	17.3	28.3
	200	320.9	433.5	16.0	24.1
	250	280.9	295.9	3.5	24.4
	300	173.3	189.7	3.9	33.6
	350	83.1	97.2	4.3	58.6
	400	43.4	53.8	7.9	107.4
	450	26.6	32.5	24.5	122.2
Al2024-TT	25	154.5	279.3	14.8	26.9
	200	107.6	158.3	10.7	57.6
	250	108.1	131.1	7.4	57.2
	300	79.6	89.1	5.3	62.3
	350	63.5	69.6	5.0	79.9
	400	44.8	48.7	5.8	91.6
	450	21.7	30.5	28.2	98.3

## Data Availability

The raw/processed data required to reproduce these findings cannot be shared at this time as the data also forms part of an ongoing study.
